# Role of Endoplasmic Reticulum-Associated Proteins in Flavivirus Replication and Assembly Complexes

**DOI:** 10.3390/pathogens8030148

**Published:** 2019-09-12

**Authors:** Hussin A. Rothan, Mukesh Kumar

**Affiliations:** Department of Biology, College of Arts and Sciences, Georgia State University, Atlanta, GA 30303, USA

**Keywords:** flavivirus, virus replication complex, endoplasmic reticulum, host factors

## Abstract

Flavivirus replication in host cells requires the formation of replication and assembly complexes on the cytoplasmic side of the endoplasmic reticulum (ER) membrane. These complexes consist of an ER membrane, viral proteins, and host proteins. Genome-wide investigations have identified a number of ER multiprotein complexes as vital factors for flavivirus replication. The detailed mechanisms of the role of ER complexes in flavivirus replication are still largely elusive. This review highlights the fact that the ER multiprotein complexes are crucial for the formation of flavivirus replication and assembly complexes, and the ER complexes could be considered as a target for developing successful broad-spectrum anti-flavivirus drugs.

## 1. Introduction 

Members of flavivirus genus are the most important arthropod-borne viruses causing disease in humans. This genus includes pathogens of public health importance including the West Nile virus (WNV), Japanese encephalitis virus (JEV), dengue virus (DENV), yellow fever virus (YFV), tick-borne encephalitis virus (TBEV), and Zika virus (ZIKV) [1,2]. Flaviviruses continue to spread and cause human disease in new areas of the world [3]. No effective therapies exist for treating individuals with flavivirus infections. The lack of specific therapeutics for flavivirus infections imparts a pressing need to identify the viral and host factors in flavivirus replication and disease outcome. 

Flaviviruses infect the host cells by binding with virus receptors on the cell membrane [4]. The direct interaction of the virus with the specific receptor induces clathrin-mediated endocytosis, a major endocytic process by which the cells uptake nutrients from the surrounding environment [5,6,7]. The acidic environment in the cellular endosomes facilitates the envelope disassembly and release viral genome, a capped, positive-sense, single-stranded, 11 kb RNA to the cytosol. Once the viral RNA binds to the ribosomes by 5′-cap structure, the translation process produces a viral polyprotein anchored to the ER membrane [4]. The viral polyprotein undergoes multi-sites cleavage by viral and cellular proteases into three structural proteins (Capsid [C], pre-membrane [prM], and Envelope [E]) and seven non-structural proteins (NS1, NS2A, NS2B, NS3, NS4A, NS4B, and NS5) [8]. Viral structural proteins construct new virions by C protein-viral RNA binding, covered with prM and E proteins [9]. Viral non-structural proteins are responsible for viral replication, attenuation of host immune response, manipulating cell structures and functions, and other yet to be known interactions with host proteins [10,11]. 

Viral proteins alter the endoplasmic reticulum (ER) membrane to generate new structures called vesicle packets (VPs), containing viral replication and assembly complexes [12]. The mechanisms underlying virus replication and assembly on the ER membrane remain to be fully understood. This review highlights the role of ER proteins in the formation of flavivirus replication and assembly complexes. To date, no antiviral drugs are available to combat infections caused by flaviviruses. Thus, targeting the host factors that have important roles in regulating the formation and stabilization of flavivirus replication and assembly complexes represents a new therapeutic approach for developing anti-flavivirus drugs.

## 2. Flavivirus Replication and Assembly Complexes

Flaviviruses replicate in host cells on the cytoplasmic side of the ER membrane. The ER membrane undergoes an extensive re-arrangement after releasing viral proteins, creating different structures. Flavivirus proteins induce a remodeling of the ER membrane into three distinct structures: vesicle packets (VPs), convoluted membranes (CM), and membrane vesicles (Ve) [13,14,15,16]. The small viral non-structural proteins serve as the scaffold for the membrane-associated replication complex [17]. NS1, NS2A, NS2B, NS4A, and NS4B are characterized as viral proteins that have the ability to remodel the ER membrane [18,19,20]. For example, ZIKV NS2A has a single segment that traverses the ER membrane and six segments that peripherally associate with the ER membrane, which are essential for viral RNA synthesis and virion assembly [21]. The formations of NS4A and NS4B homo-oligomers and hetero-oligomers and NS1 homo-dimers are necessary to remodel the ER membrane [22]. However, it is unclear how the viral proteins are repositioned in the ER-lumen or translocated to the cytoplasm during the rearrangement of the ER membrane. 

**Figure 1:**
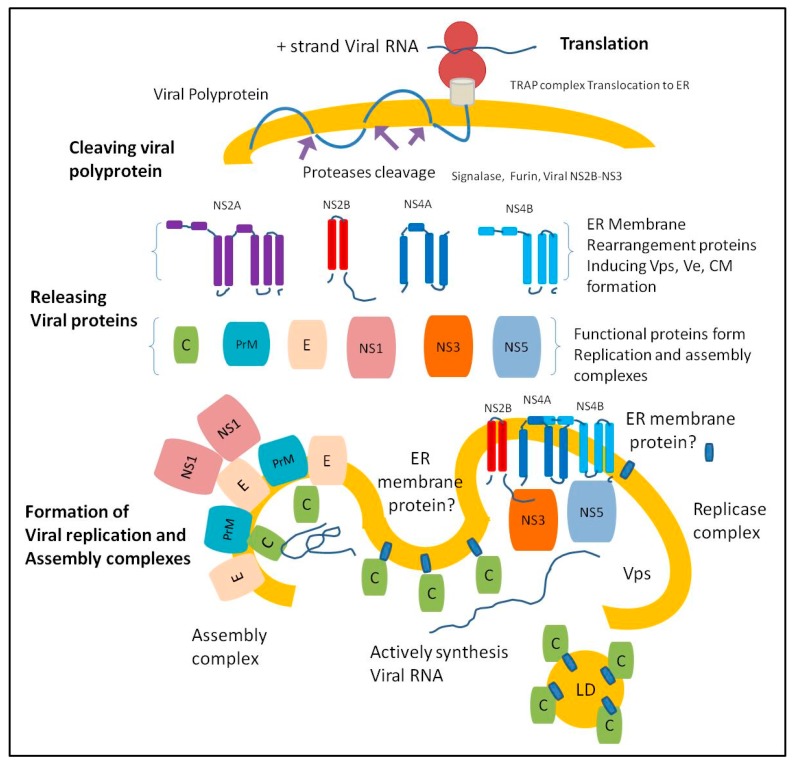
The endoplasmic reticulum (ER) membrane undergoes extensive re-arrangement after releasing viral proteins creating distinct structures. Once the ER-associated ribosome translates viral RNA, the ER complex called mammalian translocon-associated protein (TRAP) translocates the newly synthesized viral polyprotein to the ER lumen and the translocated polyprotein integrates into the ER membrane [38,39]. The cleaving of the viral polyprotein occurs when the host cell proteases (furin and signalase) gain access to the cleaving sites of the viral polyprotein, releasing viral protease units (NS2B and NS3). The central 40 amino-acid region of the NS2B co-factor is crucial to the NS3 protease function. The NS2B-NS3 serine protease cleaves viral polyprotein at various sites to release the structural and non-structural proteins [40,41,42,43,44,45,46,47].

Flavivirus replication depends on the enzymatic activities of NS3 and NS5 proteins that form a replication complex. NS3 has a protease, helicase, adenosine triphosphatase (ATPase), and RNA 5′ triphosphatase (RTPase) activities [23,24,25,26] and NS5 has methyltransferase and RNA-dependent RNA polymerase activities [27,28,29]. NS3 helicase and ATPase activities are required for unwinding double-stranded RNA utilizing the chemical energy derived from ATP hydrolysis [30]. Flavivirus capsid protein associates with the ER membrane and distributes on the surface of the lipids droplets (LDs) in the cytoplasm [31]. ER membrane-associated capsid protein distributes close to the exit of RNA replication sites (the vesicle packets) [13,16,32]. Viral NS3 plays an important role in the timing of RNA synthesis (helicase activity) and capsid protein maturation (cleave PreM-C junction as protease). The timing of capsid protein maturation and localization close to RNA synthesis are important for the capsid protein function as a RNA chaperone during virus assembly [33,34,35,36,37] (Figure 1).

## 3. ER proteins Required for Flavivirus Replication and Assembly 

CRISPR and Genomic RNA interference screens indicated that flaviviruses require overlapping as well as specific host factors to promote viral infection. The Genome-wide loss of function studies identified various ER proteins crucial for virus replication. These proteins are involved in the regulation of the stress response, protein modification and degradation, RNA translation, signal transduction, and apoptosis [48,49,50,51,52,53]. 

1. DNAJ homolog subfamily C member 14 (DNAJC14)

DNAJC14 is the heat shock protein40 (Hsp40) chaperone (also named DRIP78, Jiv, and LIP6) that modulates the dopamine D1 receptor transport from the ER to the plasma membrane [54]. DNAJC14 contains a conserved 70-amino-acid J domain that interacts with Hsp70 family members to stimulate ATP hydrolysis during chaperone activity [55]. Interestingly, the interaction between DNAJC14 and flavivirus non-structural proteins altered the properties of the ER membrane and resulted in the formation of the protein scaffold that maintains the viral replication complex [56]. Thus, DNAJC14 is a vital ER-associated chaperone required for the integration of the flavivirus replication complex to a specific ER membrane location [56,57]. Furthermore, DNAJC14 plays a central role in ER stress-associated unconventional protein secretion [58] that are induced during virus infection. It is also shown to co-localize with dsRNA within the YFV replication complex. It has been reported that endogenous levels of DNAJC14 are vital for YFV replication [57]. A similar role of DNAJC14 has recently been observed in the RNA replication of the bovine viral diarrhea virus (BVDV) [59]. Therefore, DNAJC14 is a key host cell factor for flavivirus replication.

2. Hrd1 complex 

The endoplasmic reticulum-associated degradation (ERAD) pathway includes misfolded protein recognition, translocation, ubiquitylation, and cytoplasmic proteasomal degradation. When the nascent polypeptide is synthesized by the ribosomes, it will integrate into the ER membrane and dislocate to the ER lumen [60]. ER chaperones process protein folding and assemble the subunits in functional and secreted multi-domain proteins. Viral proteins like NS3 and NS5 are multi-domain and multi-functional enzymes that may require refolding processing by cellular chaperones. Thus, the accumulation of viral proteins in the ER lumen induces the unfolded protein response (UPR) to ER stress and up-regulate cellular chaperones expression to expedite viral protein refolding [61,62]. The misfolded proteins and orphan subunits are subjected to the Hrd1 complex for translocation, ubiquitylation, and proteasomal degradation [63,64]. The Hrd1 complex consists of ER luminal lectins, chaperones, ER membrane proteins, and cytoplasmic proteins [65].

Hrd1 protein has emerged as a critical host factor required for flaviviruses replication [48,49,50,51,66,67]. The inhibition of protein translocation from ER to the cytosol or inhibiting the ER chaperone grp94 by small molecule compounds led to a significant decrease in DENV and ZIKV replication [68,69]. Blocking proteasomal degradation by the proteasome inhibitors Bortezomib significantly decreased DENV and ZIKV replication in vitro and attenuated the infection in vivo [70,71]. Genomic and proteomic screening methods have demonstrated that DENV and WNV replication requires Hrd1, Derlin2, and Ube2j1 proteins of the Hrd1 complex [48]. Proteins in the Hrd1 complex have been reported to interact with several viral proteins. For example, Derlin2 interacts with NS5 of DENV and ZIKV and with NS4B of ZIKV [51,67] (Table 1). Thus, targeting Hrd1 complex could be a new avenue in developing novel anti- flavivirus drugs [68,69].

3. Oligosaccharyltransferase (OST) complex 

The ER-associated oligosaccharyltransferase (OST) complex catalyzes the N-linked glycosylation of newly synthesized proteins. The two OST protein isoforms, which are multiprotein complexes, are composed of a catalytic subunit, STT3A or STT3B, and accessory subunits [73]. The OST complex is associated with flavivirus replication through the interaction with viral non-structural proteins. The deletion of OST subunits resulted in a >99% reduction of flavivirus infections in cell culture [50]. The catalytic function of the OST complex is not required for DENV replication, suggesting that the complex may have a structural role in the formation of the replication complex [47]. DENV RNA replication is independent on the presence of both OST isoforms, while ZIKV, YFV, and WNV replication exclusively depend on the STT3A OST complex, demonstrating differences in the requirement for OST complex variants among flaviviruses [74]. The OST complex activity is inhibited by a small molecule compound named NGI-1 [75]. This OST inhibitor exhibited anti-viral activity against flaviviruses indicating that the OST cellular pathway could be exploited for anti-viral drug discovery [74].

4. Reticulon 3 (RTN 3.1A)

In mammals, the Reticulon protein family comprises of RTN1, RTN2, RTN3, and RTN4, which are widely expressed in most tissues, especially in human and mouse brains [76,77,78]. The RTN 3.1A, ER-membrane proteins reside primarily within the ER and Golgi apparatus [76]. It is known that RTN3.1A facilitates WNV, DENV, and ZIKV replication via direct or indirect interaction with viral NS4A to facilitate replication complex formation [79]. The absence of RTN3.1A promotes the degradation of the viral NS4A protein, eventually disturbing the formation of the replication complex and the production of viral particles [79]. 

5. ER membrane complex (EMC) 

Several studies have reported the critical role of the ER membrane complex (EMC) in flavivirus replication [50,52,53]. It has been proposed that EMC serves as an ER chaperone for processing the multi-pass transmembrane proteins [80,81,82] such as flavivirus membrane proteins, NS2A, NS2B, NS4A, and NS4B, which are necessary for virus replication. It has been reported that EMC promotes the biogenesis of DENV and ZIKV non-structural multi-pass transmembrane proteins NS4A and NS4B [83]. Furthermore, EMC serves as an insertase for selective tail-anchored membrane proteins [84]. The EMC has been found to bind to NS4B and colocalized with the DENV replication organelle [83]. 

## 4. ER-Associated Proteins Critical for Virus Assembly and Egress

The role of ER protein complexes in flavivirus assembly and egress has not been extensively investigated. Nevertheless, some studies showed that individual ER proteins have a significant role in flavivirus assembly and egress. It has been reported that DDX56, a host helicase facilitates WNV assembly by transferring the newly synthesized viral RNA to the assembly site through direct binding to the capsid [85]. Other host proteins such as Src Kinases also represent crucial factors for DENV and WNV assembly and egress by facilitating virus passing from ER to the Golgi [86]. KDEL receptors (KDELR), which cycle between the ER and Golgi apparatus is vital for DENV trafficking from ER to Golgi by interacting with viral prM [87]. Other factors that are essential for DENV, WNV, and JEV assembly and egress includes Ras-related in brain protein (Rab8b), endosomal sorting complex that is required for transport (ESCRT), and the ADP-ribosylation proteins (Arf4/5). [88,89,90,91].

## 5. Conclusions

Flaviviruses exploit the ER function during infection to gain optimal replication. Multiple independent genome-wide screen studies have identified several ER-associated complexes and individual proteins that are important for flavivirus replication. These complexes such as DNAJC14, Hrd1, EMC, and RTN 3.1A play a crucial role in the construction and function of virus replication and assembly complexes. Thus, these ER- complexes represent promising host targets for developing broad-spectrum anti-flavivirus drugs. 

## Figures and Tables

**Table 1:** Hrd1 complex proteins required for flaviviruses replication.

Flavivirus	Hrd1 Subunits Required for Virus Replication	References
Dengue virus	SEL1L, AUP1, DERL2,UBE2J1, EMC2	[51,67]
West Nile virus	Hrd1, DERL2, SEL1L, UBE2J1, UBE2G2,	[48,49]
Zika virus	DERL2, AUP1	[51]
Japanese encephalitis virus	GRP78 (Bip)	[72]

## References

[1] Rice C.M. (1990). Overview of flavivirus molecular biology and future vaccine development via recombinant DNA. Southeast Asian J. Trop. Med. Public Health.

[2] Brinton M.A. (2013). Replication Cycle and Molecular Biology of the West Nile Virus. Viruses.

[3] Rothan H.A., Bidokhti M.R., Byrareddy S.N. (2018). Current concerns and perspectives on Zika virus co-infection with arboviruses and HIV. J. Autoimmun..

[4] Grove J., Marsh M. (2011). The cell biology of receptor-mediated virus entry. J. Cell Biol..

[5] Chu J.J.H., Ng M.L. (2004). Infectious Entry of West Nile Virus Occurs through a Clathrin-Mediated Endocytic Pathway. J. Virol..

[6] Sun X., Yau V.K., Briggs B.J., Whittaker G.R. (2005). Role of clathrin-mediated endocytosis during vesicular stomatitis virus entry into host cells. Virology.

[7] Blanchard E., Belouzard S., Goueslain L., Wakita T., Dubuisson J., Wychowski C., Rouillé Y. (2006). Hepatitis C Virus Entry Depends on Clathrin-Mediated Endocytosis. J. Virol..

[8] Chambers T.J., Hahn C.S., Galler R., Rice C.M. (1990). Flavivirus Genome Organization, Expression, and Replication. Annu. Rev. Microbiol..

[9] Perera R., Kuhn R.J. (2008). Structural Proteomics of Dengue Virus. Curr. Opin. Microbiol..

[10] Rothman A.L. (2011). Immunity to dengue virus: A tale of original antigenic sin and tropical cytokine storms. Nat. Rev. Immunol..

[11] Rodriguez-Madoz J.R., Belicha-Villanueva A., Bernal-Rubio D., Ashour J., Ayllon J., Fernandez-Sesma A. (2010). Inhibition of the type I interferon response in human dendritic cells by dengue virus infection requires a catalytically active NS2B3 complex. J. Virol..

[12] Neufeldt C.J., Cortese M., Acosta E.G., Bartenschlager R. (2018). Rewiring cellular networks by members of the Flaviviridae family. Nat. Rev. Genet..

[13] Welsch S., Miller S., Romero-Brey I., Merz A., Bleck C.K., Walther P., Fuller S.D., Antony C., Krijnse-Locker J., Bartenschlager R. (2009). Composition and Three-Dimensional Architecture of the Dengue Virus Replication and Assembly Sites. Cell Host Microbe.

[14] Gillespie L.K., Hoenen A., Morgan G., MacKenzie J.M. (2010). The Endoplasmic Reticulum Provides the Membrane Platform for Biogenesis of the Flavivirus Replication Complex. J. Virol..

[15] Offerdahl D.K., Dorward D.W., Hansen B.T., Bloom M.E. (2012). A Three-Dimensional Comparison of Tick-Borne Flavivirus Infection in Mammalian and Tick Cell Lines. PLoS ONE.

[16] Junjhon J., Pennington J.G., Edwards T.J., Perera R., Lanman J., Kuhn R.J. (2014). Ultrastructural Characterization and Three-Dimensional Architecture of Replication Sites in Dengue Virus-Infected Mosquito Cells. J. Virol..

[17] Zou J., Xie X., Wang Q.Y., Dong H., Lee M.Y., Kang C., Yuan Z., Shi P.Y. (2015). Characterization of Dengue Virus NS4A and NS4B Protein Interaction. J. Virol..

[18] Akey D.L., Brown W.C., Dutta S., Konwerski J., Jose J., Jurkiw T.J., DelProposto J., Ogata C.M., Skiniotis G., Kuhn R.J. (2014). Flavivirus NS1 structures reveal surfaces for associations with membranes and the immune system. Science.

[19] Paul D., Bartenschlager R. (2015). Flaviviridae Replication Organelles: Oh, What a Tangled Web We Weave. Annu. Rev. Virol..

[20] Brown W.C., Akey D.L., Konwerski J.R., Tarrasch J.T., Skiniotis G., Kuhn R.J., Smith J.L. (2016). Extended Surface for Membrane Association in Zika Virus NS1 Structure. Nat. Struct. Mol. Biol..

[21] Zhang X., Xie X., Zou J., Xia H., Shan C., Chen X., Shi P.Y. (2019). Genetic and biochemical characterizations of Zika virus NS2A protein. Emerg. Microbes Infect..

[22] Zou J., Xie X., Lee L.T., Chandrasekaran R., Reynaud A., Yap L., Wang Q.-Y., Dong H., Kang C., Yuan Z. (2014). Dimerization of Flavivirus NS4B Protein. J. Virol..

[23] Luo D., Xu T., Watson R.P., Scherer-Becker D., Sampath A., Jahnke W., Yeong S.S., Wang C.H., Lim S.P., Strongin A. (2008). Insights into RNA unwinding and ATP hydrolysis by the flavivirus NS3 protein. EMBO J..

[24] Luo D., Xu T., Hunke C., Gruber G., Vasudevan S., Lescar J. (2008). Crystal structure of the NS3 protease-helicase from dengue virus. Acta Crystallogr. Sect. A Found. Crystallogr..

[25] Luo D., Wei N., Doan D.N., Paradkar P.N., Chong Y., Davidson A.D., Kotaka M., Lescar J., Vasudevan S.G. (2010). Flexibility between the Protease and Helicase Domains of the Dengue Virus NS3 Protein Conferred by the Linker Region and Its Functional Implications. J. Biol. Chem..

[26] Bollati M., Álvarez K., Assenberg R., Baronti C., Canard B., Cook S., Coutard B., Decroly E., De Lamballerie X., Gould E.A. (2010). Structure and functionality in flavivirus NS-proteins: Perspectives for drug design. Antivir. Res..

[27] Egloff M.-P., Decroly E., Malet H., Selisko B., Benarroch D., Ferron F., Canard B. (2007). Structural and Functional Analysis of Methylation and 5′-RNA Sequence Requirements of Short Capped RNAs by the Methyltransferase Domain of Dengue Virus NS5. J. Mol. Biol..

[28] Issur M., Geiss B.J., Bougie I., Picard-Jean F., Despins S., Mayette J., Hobdey S.E., Bisaillon M. (2009). The flavivirus NS5 protein is a true RNA guanylyltransferase that catalyzes a two-step reaction to form the RNA cap structure. RNA.

[29] Bollati M., Milani M., Mastrangelo E., Ricagno S., Tedeschi G., Nonnis S., Decroly E., Selisko B., De Lamballerie X., Coutard B. (2009). Recognition of RNA Cap in the Wesselsbron Virus NS5 Methyltransferase Domain: Implications for RNA-Capping Mechanisms in Flavivirus. J. Mol. Biol..

[30] Singleton M.R., Wigley D.B. (2002). Modularity and Specialization in Superfamily 1 and 2 Helicases. J. Bacteriol..

[31] Samsa M.M., Mondotte J.A., Iglesias N.G., Assunção-Miranda I., Barbosa-Lima G., Da Poian A.T., Bozza P.T., Gamarnik A.V. (2009). Dengue Virus Capsid Protein Usurps Lipid Droplets for Viral Particle Formation. PLoS Pathog..

[32] Miorin L., Romero-Brey I., Maiuri P., Hoppe S., Krijnse-Locker J., Bartenschlager R., Marcello A. (2013). Three-Dimensional Architecture of Tick-Borne Encephalitis Virus Replication Sites and Trafficking of the Replicated RNA. J. Virol..

[33] Yamshchikov V.F., Compans R.W. (1994). Processing of the intracellular form of the west Nile virus capsid protein by the viral NS2B-NS3 protease: An in vitro study. J. Virol..

[34] Yamshchikov V.F., Compans R.W. (1993). Regulation of the Late Events in Flavivirus Protein Processing and Maturation. Virology.

[35] Amberg S.M., Nestorowicz A., McCourt D.W., Rice C.M. (1994). NS2B-3 proteinase-mediated processing in the yellow fever virus structural region: In vitro and in vivo studies. J. Virol..

[36] Markoff L., Falgout B., Chang A. (1997). A Conserved Internal Hydrophobic Domain Mediates the Stable Membrane Integration of the Dengue Virus Capsid Protein. Virology.

[37] Stocks C.E., Lobigs M. (1998). Signal Peptidase Cleavage at the Flavivirus C-prM Junction: Dependence on the Viral NS2B-3 Protease for Efficient Processing Requires Determinants in C, the Signal Peptide, and prM. J. Virol..

[38] Puschnik A.S., Majzoub K., Ooi Y.S., Carette J.E. (2017). A CRISPR toolbox to study virus–host interactions. Nat. Rev. Genet..

[39] Nagasawa K., Higashi T., Hosokawa N., Kaufman R.J., Nagata K. (2007). Simultaneous induction of the four subunits of the TRAP complex by ER stress accelerates ER degradation. EMBO Rep..

[40] Yusof R. (2000). Purified NS2B/NS3 Serine Protease of Dengue Virus Type 2 Exhibits Cofactor NS2B Dependence for Cleavage of Substrates with Dibasic Amino Acids in Vitro. J. Biol. Chem..

[41] Chambers T.J., Nestorowicz A., Amberg S.M., Rice C.M. (1993). Mutagenesis of the yellow fever virus NS2B protein: Effects on proteolytic processing, NS2B-NS3 complex formation, and viral replication. J. Virol..

[42] Falgout B., Pethel M., Zhang Y.M., Lai C.J. (1991). Both nonstructural proteins NS2B and NS3 are required for the proteolytic processing of dengue virus nonstructural proteins. J. Virol..

[43] Li H., Clum S., You S., Ebner K.E., Padmanabhan R. (1999). The Serine Protease and RNA-Stimulated Nucleoside Triphosphatase and RNA Helicase Functional Domains of Dengue Virus Type 2 NS3 Converge within a Region of 20 Amino Acids. J. Virol..

[44] Wengler G., Czaya G., Färber P.M., Hegemann J.H. (1991). In vitro synthesis of West Nile virus proteins indicates that the amino-terminal segment of the NS3 protein contains the active centre of the protease which cleaves the viral polyprotein after multiple basic amino acids. J. Gen. Virol..

[45] Assenberg R., Mastrangelo E., Walter T.S., Verma A., Milani M., Owens R.J., Stuart D.I., Grimes J.M., Mancini E.J. (2009). Crystal Structure of a Novel Conformational State of the Flavivirus NS3 Protein: Implications for Polyprotein Processing and Viral Replication. J. Virol..

[46] Rothan H.A., Han H.C., Ramasamy T.S., Othman S., Rahman N.A., Yusof R. (2012). Inhibition of dengue NS2B-NS3 protease and viral replication in Vero cells by recombinant retrocyclin-1. BMC Infect. Dis..

[47] Rothan H.A., Abdulrahman A.Y., Sasikumer P.G., Othman S., Rahman N.A., Yusof R. (2012). Protegrin-1 Inhibits Dengue NS2B-NS3 Serine Protease and Viral Replication in MK2 Cells. J. Biomed. Biotechnol..

[48] Krishnan M.N., Ng A., Sukumaran B., Gilfoy F.D., Uchil P.D., Sultana H., Brass A.L., Adametz R., Tsui M., Qian F. (2008). RNA interference screen for human genes associated with West Nile virus infection. Nature.

[49] Ma H., Dang Y., Wu Y., Jia G., Anaya E., Zhang J., Abraham S., Choi J.G., Shi G., Qi L. (2015). A CRISPR-based screen identifies genes essential for West Nile virus-induced cell death. Cell Rep..

[50] Marceau C.D., Puschnik A.S., Majzoub K., Ooi Y.S., Brewer S.M., Fuchs G., Swaminathan K., Mata M.A., Elias J.E., Sarnow P. (2016). Genetic dissection of Flaviviridae host factors through genome-scale CRISPR screens. Nature.

[51] Scaturro P., Stukalov A., Haas D.A., Cortese M., Draganova K., Płaszczyca A., Bartenschlager R., Götz M., Pichlmair A. (2018). An orthogonal proteomic survey uncovers novel Zika virus host factors. Nature.

[52] Savidis G., McDougall W.M., Meraner P., Perreira J.M., Portmann J.M., Trincucci G., John S.P., Aker A.M., Renzette N., Robbins D.R. (2016). Identification of Zika Virus and Dengue Virus Dependency Factors using Functional Genomics. Cell Rep..

[53] Zhang R., Miner J.J., Gorman M.J., Rausch K., Ramage H., White J.P., Zuiani A., Zhang P., Fernandez E., Zhang Q. (2016). A CRISPR screen defines a signal peptide processing pathway required by flaviviruses. Nature.

[54] Bermak J.C., Li M., Bullock C., Zhou Q.-Y. (2001). Regulation of transport of the dopamine D1 receptor by a new membrane-associated ER protein. Nature.

[55] Vos M.J., Hageman J., Carra S., Kampinga H.H. (2008). Structural and Functional Diversities between Members of the Human HSPB, HSPH, HSPA, and DNAJ Chaperone Families. Biochemisty.

[56] Yi Z., Yuan Z., Rice C.M., Macdonald M.R. (2012). Flavivirus Replication Complex Assembly Revealed by DNAJC14 Functional Mapping. J. Virol..

[57] Yi Z., Sperzel L., Nürnberger C., Bredenbeek P.J., Lubick K.J., Best S.M., Stoyanov C.T., Law L.M.J., Yuan Z., Rice C.M. (2011). Identification and Characterization of the Host Protein DNAJC14 as a Broadly Active Flavivirus Replication Modulator. PLoS Pathog..

[58] Jung J., Kim J., Roh S.H., Jun I., Sampson R.D., Gee H.Y., Choi J.Y., Lee M.G. (2016). The HSP70 co-chaperone DNAJC14 targets misfolded pendrin for unconventional protein secretion. Nat. Commun..

[59] Isken O., Postel A., Bruhn B., Lattwein E., Becher P., Tautz N. (2019). CRISPR/Cas9-Mediated Knockout of DNAJC14 Verifies This Chaperone as a Pivotal Host Factor for RNA Replication of Pestiviruses. J. Virol..

[60] Ghaemmaghami S., Huh W.K., Bower K., Howson R.W., Belle A., Dephoure N., O’Shea E.K., Weissman J.S.J.S., Howson R.W., O’Shea E.K.E.K. (2003). Global analysis of protein expression in yeast. Nature.

[61] Lee Y.R., Kuo S.H., Lin C.Y., Fu P.J., Lin Y.S., Yeh T.M., Liu H.S. (2018). Dengue virus-induced ER stress is required for autophagy activation, viral replication, and pathogenesis both in vitro and in vivo. Sci. Rep..

[62] Blázquez A.B., Escribano-Romero E., Merino-Ramos T., Saiz J.C., Martín-Acebes M.A. (2014). Stress responses in flavivirus-infected cells: Activation of unfolded protein response and autophagy. Front. Microbiol..

[63] Christianson J.C., Olzmann J.A., Shaler T.A., Sowa M.E., Bennett E.J., Richter C.M., Tyler R.E., Greenblatt E.J., Harper J.W., Kopito R.R. (2011). Defining human ERAD networks through an integrative mapping strategy. Nature..

[64] Olzmann J.A., Kopito R.R., Christianson J.C. (2013). The mammalian endoplasmic reticulum-associated degradation system. Cold Spring Harb. Perspect. Biol..

[65] Hampton R.Y., Gardner R.G., Rine J. (1996). Role of 26S proteasome and HRD genes in the degradation of 3-hydroxy-3-methylglutaryl-CoA reductase, an integral endoplasmic reticulum membrane protein. Mol. Biol. Cell.

[66] Viktorovskaya O.V., Greco T.M., Cristea I.M., Thompson S.R. (2016). Identification of RNA Binding Proteins Associated with Dengue Virus RNA in Infected Cells Reveals Temporally Distinct Host Factor Requirements. PLoS Negl. Trop. Dis..

[67] Mairiang D., Zhang H., Sodja A., Murali T., Suriyaphol P., Malasit P., Limjindaporn T., Finley R.L. (2013). Identification of New Protein Interactions between Dengue Fever Virus and Its Hosts, Human and Mosquito. PLoS ONE.

[68] Ruan J., Rothan H.A., Zhong Y., Yan W., Henderson M.J., Chen F., Fang S. (2019). A small molecule inhibitor of ER-to-cytosol protein dislocation exhibits anti-dengue and anti-Zika virus activity. Sci. Rep..

[69] Rothan H.A., Zhong Y., Sanborn M.A., Teoh T.C., Ruan J., Yusof R., Hang J., Henderson M.J., Fang S. (2019). Small molecule grp94 inhibitors block dengue and Zika virus replication. Antivir. Res..

[70] Choy M.M., Zhang S.L., Costa V.V., Tan H.C., Horrevorts S., Ooi E.E. (2015). Proteasome Inhibition Suppresses Dengue Virus Egress in Antibody Dependent Infection. PLoS Negl. Trop. Dis..

[71] Barrows N.J., Campos R.K., Powell S.T., Prasanth K.R., Schott-Lerner G., Soto-Acosta R., Galarza-Muñoz G., McGrath E.L., Urrabaz-Garza R., Gao J. (2016). A screen of FDA-approved drugs for inhibitors of Zika virus infection. Cell Host Microbe.

[72] Nain M., Mukherjee S., Karmakar S.P., Paton A.W., Paton J.C., Abdin M.Z., Basu A., Kalia M., Vrati S. (2017). GRP78 Is an Important Host Factor for Japanese Encephalitis Virus Entry and Replication in Mammalian Cells. J. Virol..

[73] Shrimal S., Cherepanova N.A., Gilmore R. (2015). Cotranslational and posttranslocational N-glycosylation of proteins in the endoplasmic reticulum. Semin. Cell Dev. Biol..

[74] Puschnik A.S., Marceau C.D., Ooi Y.S., Majzoub K., Rinis N., Contessa J.N., Carette J.E. (2017). A small molecule oligosaccharyltransferase inhibitor with pan-flaviviral activity. Cell Rep..

[75] Lopez-Sambrooks C., Shrimal S., Khodier C., Flaherty D.P., Rinis N., Charest J.C., Gao N., Zhao P., Wells L., Lewis T.A. (2016). Oligosaccharyltransferase Inhibition Induces Senescence in RTK-Driven Tumor Cells. Nat. Methods.

[76] Kume H., Konishi Y., Murayama K.S., Kametani F., Araki W. (2009). Expression of reticulon 3 in Alzheimer’s disease brain. Neuropathol. Appl. Neurobiol..

[77] Cai Y., Saiyin H., Lin Q., Zhang P., Tang L., Pan X., Yu L. (2005). Identification of a new RTN3 transcript, RTN3-A1, and its distribution in adult mouse brain. Mol. Brain Res..

[78] Diekmann H., Klinger M., Oertle T., Heinz D., Pogoda H.M., Schwab M.E., Stuermer C.A.O. (2005). Analysis of the Reticulon Gene Family Demonstrates the Absence of the Neurite Growth Inhibitor Nogo-A in Fish. Mol. Biol. Evol..

[79] Aktepe T.E., Liebscher S., Prier J.E., Simmons C.P., MacKenzie J.M. (2017). The Host Protein Reticulon 3.1A Is Utilized by Flaviviruses to Facilitate Membrane Remodelling. Cell Rep..

[80] Jonikas M.C., Collins S.R., Denic V., Oh E., Quan E.M., Schmid V., Weibezahn J., Schwappach B., Walter P., Weissman J.S. (2009). Comprehensive characterization of genes required for protein folding in the endoplasmic reticulum. Science.

[81] Shurtleff M.J., Itzhak D.N., A Hussmann J., Oakdale N.T.S., A Costa E., Jonikas M., Weibezahn J., Popova K.D., Jan C.H., Sinitcyn P. (2018). The ER membrane protein complex interacts cotranslationally to enable biogenesis of multipass membrane proteins. eLife.

[82] Satoh T., Ohba A., Liu Z., Inagaki T., Satoh A.K. (2015). dPob/EMC is essential for biosynthesis of rhodopsin and other multi-pass membrane proteins in Drosophila photoreceptors. eLife.

[83] Lin D.L., Inoue T., Chen Y.J., Chang A., Tsai B., Tai A.W. (2019). The ER Membrane Protein Complex Promotes Biogenesis of Dengue and Zika Virus Non-structural Multi-pass Transmembrane Proteins to Support Infection. Cell Rep..

[84] Guna A., Volkmar N., Christianson J.C., Hegde R.S. (2018). The ER membrane protein complex is a transmembrane domain insertase. Science.

[85] Xu Z., Hobman T.C. (2012). The helicase activity of DDX56 is required for its role in assembly of infectious West Nile virus particles. Virology.

[86] Hirsch A.J., Medigeshi G.R., Meyers H.L., DeFilippis V., Früh K., Briese T., Lipkin W.I., Nelson J.A. (2005). The Src Family Kinase c-Yes Is Required for Maturation of West Nile Virus Particles. J. Virol..

[87] Li M.Y., Grandadam M., Kwok K., Lagache T., Siu Y.L., Zhang J.S., Sayteng K., Kudelko M., Qin C.F., Olivo-Marin J.C. (2015). KDEL Receptors Assist Dengue Virus Exit from the Endoplasmic Reticulum. Cell Rep..

[88] Kudelko M., Brault J.B., Kwok K., Li M.Y., Pardigon N., Peiris J.M., Bruzzone R., Desprès P., Nal B., Wang P.G. (2012). Class II ADP-ribosylation factors are required for efficient secretion of dengue viruses. J. Biol. Chem..

[89] Farhat R., Séron K., Ferlin J., Fénéant L., Belouzard S., Goueslain L., Dubuisson J., Rouillé Y., Jackson C.L. (2016). Identification of class II ADP-ribosylation factors as cellular factors required for hepatitis C virus replication. Cell. Microbiol..

[90] Kobayashi S., Suzuki T., Kawaguchi A., Phongphaew W., Yoshii K., Iwano T., Harada A., Kariwa H., Orba Y., Sawa H. (2016). Rab8b Regulates Transport of West Nile Virus Particles from Recycling Endosomes. J. Biol. Chem..

[91] Tabata K., Arimoto M., Arakawa M., Nara A., Saito K., Omori H., Arai A., Ishikawa T., Konishi E., Suzuki R. (2016). Unique Requirement for ESCRT Factors in Flavivirus Particle Formation on the Endoplasmic Reticulum. Cell Rep..

